# COGRIMEN: Coarse-Grained Method for Modeling of Membrane Proteins in Implicit Environments

**DOI:** 10.1021/acs.jctc.2c00140

**Published:** 2022-08-23

**Authors:** Przemysław Miszta, Paweł Pasznik, Szymon Niewieczerzał, Krzysztof Młynarczyk, Sławomir Filipek

**Affiliations:** † Faculty of Chemistry, Biological and Chemical Research Centre, University of Warsaw, Warsaw 02-093, Poland

## Abstract

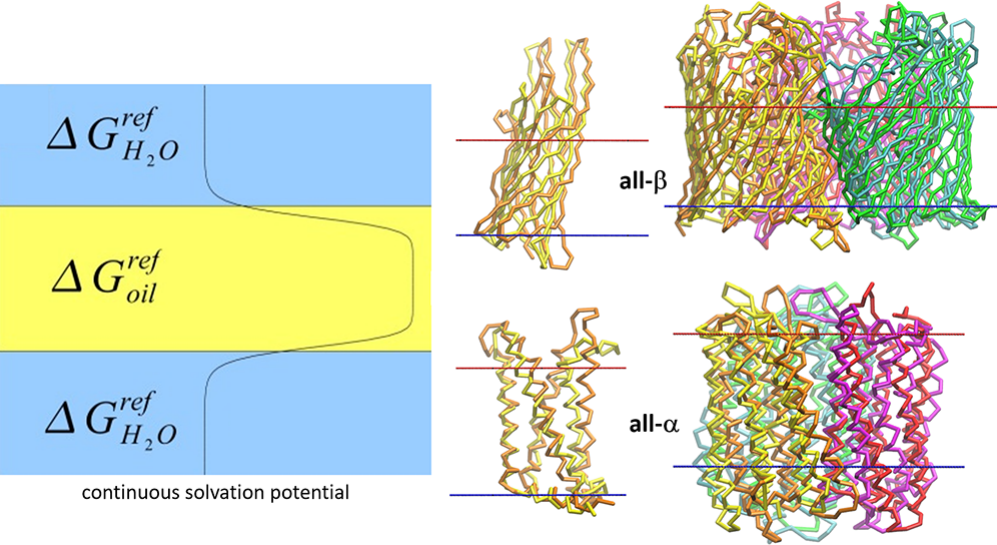

The presented methodology is based on coarse-grained representation of biomolecules in implicit environments and is designed for the molecular dynamics simulations of membrane proteins and their complexes. The membrane proteins are not only found in the cell membrane but also in all membranous compartments of the cell: Golgi apparatus, mitochondria, endosomes and lysosomes, and they usually form large complexes. To investigate such systems the methodology is proposed based on two independent approaches combining the coarse-grained MARTINI model for proteins and the effective energy function to mimic the water/membrane environments. The latter is based on the implicit environment developed for all-atom simulations in the IMM1 method. The force field solvation parameters for COGRIMEN were initially calculated from IMM1 all-atom parameters and then optimized using Genetic Algorithms. The new methodology was tested on membrane proteins, their complexes and oligomers. COGRIMEN method is implemented as a patch for NAMD program and can be useful for fast and brief studies of large membrane protein complexes.

## Introduction

The majority of currently used approaches for molecular dynamics (MD) simulations of the membrane proteins, both all-atom and coarse-grained, require one to build the membrane, stabilize it via relaxation procedures, insert a membrane protein, relax the whole system, and then run the productive simulations.^1−3^ Such an approach is time-consuming and requires expert knowledge. There is a lack of a simple methodology for novice and intermediate users who want to conduct simulations of large systems involving membrane proteins, which are still difficult to study. About 20%–30% of genes included in the human genome encode membrane proteins, so this is a large scientific area for investigations.^4^ Furthermore, the MD simulations are becoming increasingly valuable to study the membrane proteins since they can reveal the dynamic behavior of biological systems not seen in the static structures.

### Advantages of Using Coarse-Grained (CG) Methods

The atomistic simulations can provide valuable information on structural and dynamical properties of investigated systems, but to simulate collective effects such as the formation of large complexes of many membrane protein, very large membrane patches are necessary, which can only be realized using the coarse-grained (CG) methodology.^5^ Currently, the CG methods are under active development and numerous new methods are continuously emerging. However, it is worth noting that it is impossible to simultaneously represent the structure and the key thermodynamic properties of the system with pair potentials, which is known as the representability problem.^6^ The solution is to select an appropriate method to answer the current question. The main reason to use the coarse-grained modeling is that it provides a significant speedup when compared to classical all-atom MD simulations. The CG simulations allow the study of large biological systems by using the simplified but reasonable models able to reproduce the key experimental data. The idea behind the CG methods is to represent a group of atoms as one united bead and to use a longer integration time step, which enables researchers to study the behavior of the system over extended periods of time.^7−11^

The first idea of reducing the amino acid representation by grouping of adjacent atoms into a bead called a pseudoatom was based on the uniaxial Gay–Berne model.^12,13^ This approach was further developed by grouping each carbon with its bonded hydrogen atoms into one united atom.^14^ Precisely, an aliphatic carbon atom and attached hydrogen atoms were represented as one bead. The united-atom representation is widely used because it is computationally efficient and provides results in reasonable agreement with available experimental data. The idea of united atoms was further extended by coarse-grained force fields in which several heavy atoms were mapped onto one bead. Currently, the most commonly used coarse-grained force field is MARTINI,^15−17^ which is implemented in GROMACS^18,19^ and NAMD^20,21^ programs for MD simulations. In some coarse-grained methods, the implicit solvent is used instead of water beads, like in UNRES^22,23^ and CABS,^24^ and such a simplification leads to reduction of the system by at least 1 order of magnitude. Representing each amino acid, containing on average 20 atoms, by two beads reduces the number of particles in proteins by a factor of 10. If we consider large systems, calculation of forces scales proportionally to the number of particles squared, so the acceleration may by even of 2 orders of magnitude. The second factor of the speedup is the time step, which is dependent on the fastest frequencies of atomic motions, which are about 10 times slower in CG representation than in the all-atom model so the time step is proportionally larger. Another source of speedup has its origin in a fact that the energy landscape is much smoother and reduces the number of local energy minima that are present in the case of all-atom molecular dynamics. This assessment of the possible speedup is very simplified and finally depends on the application of a particular coarse-grained method and the investigated system.

### The Implicit Solvent Methodology

The lipid composition of biological membranes is variable and is known to depend on a number of factors like (a) area of the cell membrane, (b) cell type, (c) cell age, (d) environment, (e) organelle or (f) the organism.^25,26^ The stunning resemblance between the types of structures of membrane proteins present in various organisms (a bundle of α-helices or a β-barrel) contrasts with the variable nature of membrane composition. That suggests that in general the membrane proteins are tolerant to a certain extent of differences in bilayer composition.^27^ For instance G-protein-coupled receptors (GPCRs), heterologously expressed in the cells of evolutionarily distant organisms, may retain their activity (e.g., the cannabinoid receptor in bacterial cells)^28^ despite the fact that the bilayer is lacking cholesterol, which is thought to be indispensable for GPCR function.^29^ Such experimental data justifies the usage of a simple, consisting of one phospholipid type, or even the implicit membrane models in MD simulations.

The speed of calculations makes the implicit solvent models useful for fast simulations of protein systems. In 1999 Lazaridis and Karplus developed a Gaussian solvent-exclusion model for calculations of the solvation free energy. It was combined with the CHARMM19 united atom force field, to provide an effective energy function (EEF1)^30^ for proteins in water solution. Then, in 2003, Lazaridis made an extension of the EEF1 energy function to include the membranous environment. The extension consisted of (i) development of solvation parameters for united atoms in the membranous phase, (ii) introduction of a heterogeneous membrane–aqueous system by making the reference solvation free energy of each atom dependent on the *z*-axis coordinate, which is perpendicular to the membrane, (iii) introduction of the distance dependent dielectric model to account for the reduced screening of electrostatic interactions in the membrane, and (iv) an adjustment of the EEF1 aqueous parameters. The resulting IMM1 method^31^ parameters were based on calculations of the potential of mean force between amino acid side-chains in water, and experimental data for the transfer of amino acid side-chains from water to cyclohexane.

The other, simplified methodologies were created as well. The mixed atomistic and coarse-grained force field (PACSAB) was developed by Emperador et al.^32^ It uses the pairwise additive potential for coarse-grained side chains and the atomistic backbone protein model. PACSAB is a CG protein model based on an implicit solvent approach, which uses CG representation of the amino acid side chains, while keeping an atomistic representation of the backbone in order to describe accurately secondary structure elements. After the refinement^33^ PACSAB was used to study the protein aggregation and protein–protein recognition in an aqueous environment. Recently, the model was also used to study the effect of a helical structure on the ubiquitin dimerization and the conformational ensemble of the disordered protein activator for hormone and retinoid receptors.^34^ Instead of the standard MD, a discrete molecular dynamics algorithm (DMD)^35^ is employed, which allows one to use discretized interaction potentials for efficient sampling of large protein systems. The DMD algorithm has been successfully used to study protein–protein flexible docking^36^ and a simulation of conformational transition pathways in proteins.^37^ The PACSAB/DMD method accurately reproduces the aggregation properties providing images of protein ensembles exhibiting a folded core and an intrinsically disordered region.

Another approach, named the Implicit Solvation using the Superposition Approximation (IS-SPA),^38^ was used to study the molecular aggregation. It was demonstrated that the nonpolar component of the solvation force can be captured implicitly using the IS-SPA approach, which is based on the Kirkwood superposition approximation to estimate the mean force of the solvent from solute parameters. A parabolic first solvation shell was introduced for fitting the water distributions around a molecule and the Monte Carlo integration of the mean solvent force. The accuracy and transferability of the approach was demonstrated by its ability to capture the position and relative energies of a desolvation barrier and free energy minimum of alkane homodimers. The method offers a 2 orders of magnitude speedup for dilute systems as compared to explicit solvent simulations.

A large problem with the implicit solvent models is that they lack certain physical properties compared to explicit solvent models, e.g., the many-body effects of the neglected solvent molecules, which are difficult to model as a mean field. Among various attempts, for proper parametrization of CG force fields for usage in implicit aqueous systems, the machine learning approaches were also employed. ISSNet, a graph neural network, was used to model the implicit solvent potential of the mean force.^39^ It is a continuation of the previous machine learning CG models, CGnet^40^ and CGSchNet,^41^ the latter is based on a graph neural network architecture SchNet.^42^ ISSNet can use explicit solvent simulation data to compare the solute conformational distributions under different solvation treatments. The results indicate that ISSNet models can outperform the generalized Born and the surface area models in reproducing the thermodynamics of small protein systems with respect to explicit solvent. It also demonstrates the great potential of applying machine learning methods for accurate modeling of solvent effects.

The methods combining CG representation and the implicit membrane can be used to study currently unresolved problems requiring long scales of time and length like crowding of proteins in the membrane and its surroundings. In the implicit membrane the real crowding can be studied by using hundreds of copies of proteins which is currently not feasible because of large number of beads representing lipid bilayer and water. The use of such methodologies could allow simulation times to be extended and the number of protein molecules in the system to be increased, which would be beneficial in studying large membranous systems.

### Overview of the COGRIMEN Method

In the COGRIMEN method, we employ the MARTINI^17^ force field to represent coarse-grained proteins. MARTINI proved to be very effective in studying membrane behavior and lipid–protein interactions,^43^ which can be even more accurate in refined MARTINI3.^44^ In standard MARTINI, the hydrophobic effect is modeled by having stronger pairwise interactions between similar polar (P-type) and apolar (C-type) bead types compared to their cross interactions. MARTINI was extensively used to study membrane proteins and their interactions in the membrane, e.g., for SARS-CoV and SARS-CoV-2 envelopes with many spike proteins to study flexibility of these proteins,^45^ aggregation of membrane proteins with modification of MARTINI force field to address the excessive aggregation,^46^ and dimerization of GPCRs.^47^

In Dry MARTINI,^48^ the removal of the aqueous phase had to be somehow compensated with other interactions in the force field. Instead of introducing a specific term to account for solvation effects, in Dry MARTINI the strength of existing pairwise Lennard-Jones (LJ) interactions was adjusted to retain the hydrophobic/hydrophilic behavior of molecules in standard MARTINI. The other strategy was employed in COGRIMEN, being an extension of the IMM1 all-atom methodology toward CG representation of proteins, since we employed the solvation parameters. The initial values of these parameters resulted from adding the corresponding parameters of atoms from which the beads are made. Then, the Genetic Algorithm (GA) was used to optimize the solvation parameters for all beads for both the water and the membrane environments. The fitness function for the GA was based on MD simulations of nine proteins in the training set, representing mostly the membrane proteins, including three trimers. The proteins represented all-α, all-β and mixed architectures. The protein trimers, together with complexes of GPCRs with their effector proteins, were simulated for 10 μs to study their stability in order to assess the method. Based on obtained results and speed of calculations, the COGRIMEN can be useful for fast and brief studies of large membrane protein complexes.

Recently, we used the all-atom IMM1 methodology inside the Web server GPCRsignal.^49^ The server is used for dynamical analysis of the interface between GPCRs and their effector proteins, i.e., G proteins and arrestins. GPCRsignal provides a possibility of running MD simulations of currently available GPCR-effector protein complexes with the user defined mutations. The implementation of COGRIMEN and testing it using GPCR complexes enables the use of COGRIMEN to study GPCR oligomers and the phenomena of crowding of such complexes.

## Methods

### The Coarse-Grained Model

**Figure 1:**
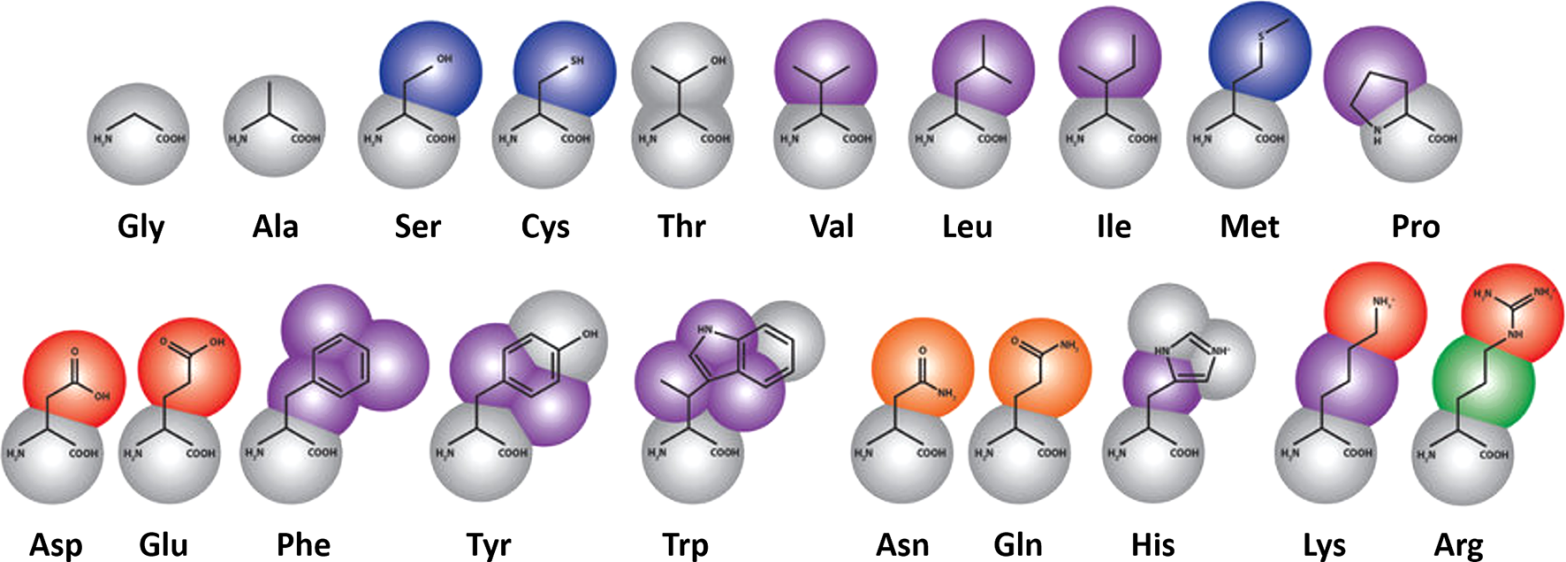
Coarse-grained mapping of amino acids according to MARTINI^17^ force field (color denotes properties of grains: purple, apolar; blue and green, intermediate; gray and orange, polar; red, charged). Figure based on Figure 3 from ref (50).

The presented methodology is based on two independent approaches to study complex biological systems. We combine the CG model of proteins and the effective energy function (EEF1) for proteins in solution^30^ as well as the implicit membrane model (IMM1).^31^ The CG model is the MARTINI force field model of Marrink et al.^15,16^ with its extension to proteins.^17^ The MARTINI model is residue-based, which means that the parameters of each bead are adjusted to reflect physical properties of a group of atoms it replaces. An average bead represents ten atoms (Figure 1), i.e., the amino acids are represented by two sites on average (one for backbone and one for side chain), with an exception of glycine and alanine which are represented by only one bead. In the standard MARTINI model there are also water beads representing four water molecules each.

For the COGRIMEN method, we employed all bonded interactions (bonds, flat angles, dihedrals) from the standard MARTINI model without modification. Standard nonbonded interactions are based on van der Waals interactions via Lennard-Jones potential and the electrostatic interactions via Coulomb potential. Van der Waals parameters for beads were taken from standard MARTINI model. In the COGRIMEN method, we remove explicit membrane and water by introducing continuous solvation potential and modify the electrostatic interactions.

### Implementation of the Solvent–Membrane Medium

The solvation term in COGRIMEN (similarly to the EEF1 method)^30^ is calculated by a combination of experimental knowledge and theoretical considerations. It is based on reference solvation parameters, Δ*G*^ref^ (the solvation of reference molecule) and takes into account a burial of the group (eq 1).
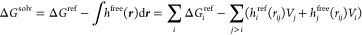
1where the integral is a solvation correction due to the presence of additional surrounding groups. Function *h*^free^(***r***) is the solvation free energy density at point ***r***. *r*_*ij*_ is the distance between beads *i* and *j* while *V*_*i*_ is a solvation volume of a particular bead and is treated as a parameter of the force field. The solvation free energy of a given conformation of the molecule can be written as an integral over the space around it. It contains contributions from solute–solvent energy, solvent reorganization energy, solute–solvent entropy, and solvent reorganization entropy. Its magnitude is largest close to the solute and decays to zero far from the solute. This function is approximated by the Gaussian distribution and is dependent on solvation parameters (Δ*G*_*i*_^free^, λ_*i*_, *R*_*i*_) for each bead type (eq 2). *R*_*i*_ is defined as a radius of a bead based on van der Waals radii of atoms included in the bead.
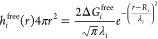
2

Since the solvent electrostatic screening is not explicitly included in the solvent-exclusion model, the distance-dependent dielectric constant is used in a form ε = *r* (in a water environment). We mimic the solvent and membrane environments by employing the IMM1 method^31^ developed for all-atom systems, where the appropriate parameters were modified in order to reflect the physicochemical properties of CG beads. According to the IMM1 model, the membrane is parallel to the *XY* plane and centered at *z* = 0. The implicit membrane is modeled by applying the additional reference solvation terms for each bead *i* (eq 3).
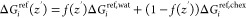
3where *z*′ = |*z*|/(*T*/2). Parameter *T* describes thickness of the hydrophobic part of the membrane. It is assumed that the interior of the membrane is a nonpolar solvent (chex: cyclohexane), and near the bilayer interface a smooth transition occurs so a pure solvent is restored beyond the membrane’s border. The reference solvation energy depends on an absolute position and a switching function (eq 4).
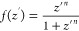
4

**Figure 2:**
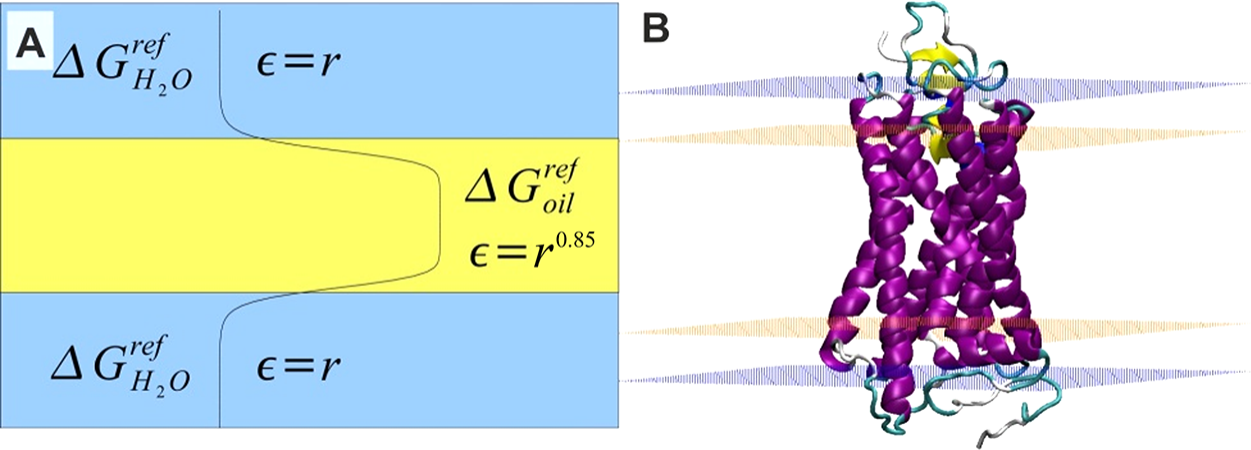
An implicit solvent method IMM1. (A) A continuous change of solvation potential in a water-membrane system. (B) A rhodopsin molecule simulated in the implicit membrane environment. Pink surfaces denote pure hydrophobic part of the membrane, blue surfaces denote bulk water areas, while the space between them corresponds to the transition area. Reproduced from Reference (1) by permission from Springer Nature Customer Service Centre GmbH: Latek, D.; Trzaskowski, B.; Niewieczerzal, S.; Miszta, P.; Mlynarczyk, K.; Debinski, A.; Pulawski, W.; Yuan, S.; Sztyler, A.; Orzel, U.; Jakowiecki, J.; Filipek, S. Modeling of Membrane Proteins. In *Computational Methods to Study the Structure and Dynamics of Biomolecules and Biomolecular Processes*; Liwo, A., Ed.; 2019; Vol. 8, pp 371–451. Copyright 2014, Springer Nature.

This function assures a transition between interior of the membrane and the pure water, while *n* controls the range of *z* where the transition between both environments occurs. *T* and *n* are characteristic for a particular type of membrane. Following the original method, we assumed *n* = 10, which gives almost complete change of environment within a range of 6 Å. This function assures a transition between interior of the membrane and a pure water. The value 10 for *n* gives the appropriate steepness of the transition between nonpolar and polar environments.^31^ At the water-membrane interface *f* = 0.5, while far from the membrane it is equal to 1. The properties of a solvent, a solvation free energy and a dielectric constant, are smoothly changing perpendicularly to the membrane (Figure 2). The same beads have different solvation parameters for bulk water and bulk membrane. In the transition phase their properties are changing smoothly so the interactions between beads are dependent on where they are located in relation to the membrane.

The electrostatic interactions in the applied CG model are present only between beads representing charged groups, and the vast majority of the beads has effective charge equal to zero. Again, based on the IMM1 method, we introduce a dielectric constant as a function of a distance between interacting sites *i* and *j* and it is defined as follows
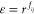
5where
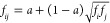
6*f*_*i*_ and *f*_*j*_ are relative positions of beads *i* and *j* and are defined by eq 4. We set *a* = 0.85, consistently with the original IMM1 model. The exponent 0.85 was found by Lazaridis^31^ to give reasonable insertion energies for various polypeptide alpha-helices. The favorable aliphatic solvation change dominates the unfavorable polar solvation change. This finding shows that to reproduce the experiment, it is necessary to include the strengthening of electrostatic interactions in the membrane. The solvation parameters required to be assessed for all beads in MARTINI, for water and membrane environments, are the bead volume *V*_*i*_, the correlation length λ_*i*_, Δ*G*_*i*_^ref^, and Δ*G*_*i*_^free^. The Δ*G*_*i*_^ref^ and Δ*G*_*i*_^free^ values refer to 298 K. Values at other temperatures are determined using Δ*H*_*i*_^ref^ and Δ*c*_*pi*_^ref^, and these parameters were calculated by adding the appropriate atomic parameters for each bead. They were not adjusted because we did not conduct simulations in temperatures other than 298 K. The bead parameters: *V*_*i*_, λ_*i*_, Δ*G*_*i*_^ref^, and Δ*G*_*i*_^free^, were fine-tuned using a GA procedure based on structural features derived from MD simulations of a training set of proteins.

### Selection of Proteins for Development of Solvation Parameters

**Figure 3:**
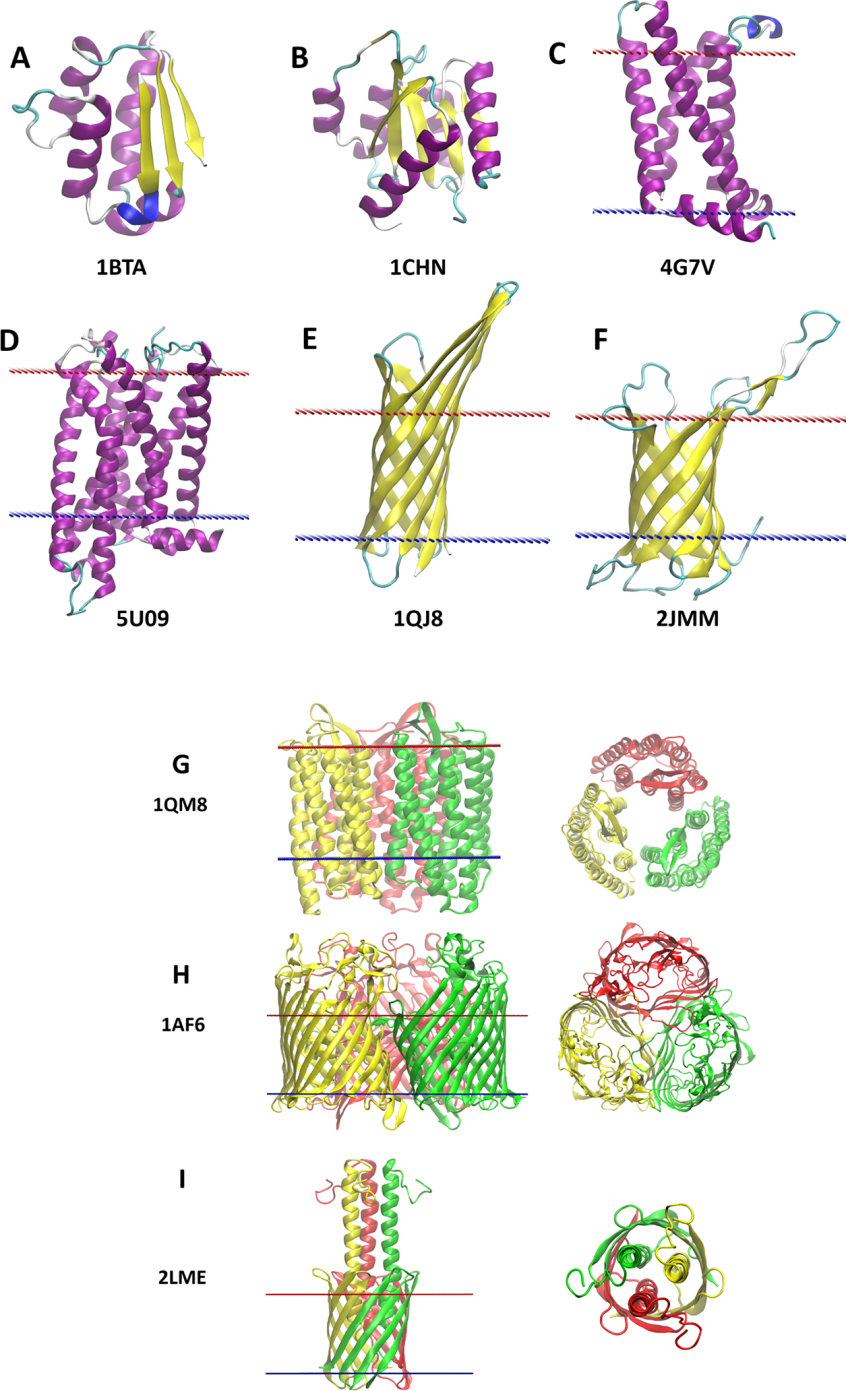
Training set of proteins taken for development of solvation parameters with their PDB IDs. (A) barstar protein; (B) bacterial chemotaxis protein CheY; (C) 4TM isolated voltage-sensing domain; (D) 7TM CB1 cannabinoid receptor; (E) the outer membrane protein OmpX; (F) the outer membrane protein OmpA; (G) trimer of bacteriorhodopsin from *Halobacterium salinarum*; (H) trimer of maltoporin from *Escherichia coli*; (I) a trimer composed of membranous β-barrel and the extramembrane helical bundle of anchor protein from *Yersinia enterocolitica*. Colors in panels A–F denote the secondary elements in particular proteins. Colors in panels G–I denote particular monomers. For membrane proteins the membrane is marked by red and blue dotted surfaces. The membrane thicknesses for individual proteins and complexes were taken from Orientations of Proteins in Membranes (OPM) database.^60^

To perform CG MD simulations in implicit environments, the solvation parameters should be developed for the membranous and the water implicit environments. We selected nine proteins representing both environments: two αβ small cytoplasmic proteins (Figure 3A,B), two all-α (Figure 3C,D) and two all-β (Figure 3E,F) transmembrane proteins, as well as three transmembrane trimers: all-α, all-β, and mixed (Figure 3G,H,I). The two cytoplasmic proteins were barstar protein from *Bacillus amyloliquefaciens* (PDB id: 1BTA)^51^ and bacterial chemotaxis protein CheY from *Escherichia coli* (PDB id: 1CHN).^52^ Among the transmembrane proteins there were two all-helical proteins: the isolated voltage-sensing domain from *Ciona intestinalis* (PDB id: 4G7V)^53^ and human CB1 cannabinoid receptor (PDB id: 5U09),^54^ as well as two β-barrel proteins from *Escherichia coli*: the outer membrane protein OmpX (PDB id: 1QJ8)^55^ and OmpA (PDB id: 2JMM).^56^ We also used trimeric membranous systems: all-helical protein bacteriorhodopsin from *Halobacterium salinarum* (PDB id: 1QM8),^57^ a trimer of β-barrel protein maltoporin from *Escherichia coli* (PDB id: 1AF6),^58^ and a trimeric anchor protein from *Yersinia enterocolitica* (PDB id: 2LME) being a hybrid composed of a membranous β-barrel and the extramembrane α-helical bundle.^59^

### Preparation of Proteins for MD Simulations

Apart from proteins in the training set, we also prepared two complexes of GPCRs with effector proteins as an additional, external test of COGRIMEN. Two complexes were selected for this purpose: a complex of β2-adrenergic receptor with a Gs trimer (PDB id: 3SN6)^61^ and a complex of rhodopsin with arrestin (PDB id: 4ZWJ).^62^ The fusion protein, lysozyme T4, as well as nonpeptide ligands were removed from both complexes. The same was done for the training set of proteins: the fusion protein and a ligand were removed from the CB1 receptor structure, and all ligands were removed from other proteins from the training set. To keep the protein integrity the fragments of loops not visible in the crystal structures were rebuilt using the BuildLoop function in the YASARA Structure v.20.12 program.^63^ Loops were constructed by searching a nonredundant set of the Protein Data Bank (PDB) (90% sequence identity cutoff, resolution better than 2.5 Å). The loops have been built in so as not to adversely affect the covalent geometry around the anchor points.^64^ N- and C-termini not visible in the crystal were not restored. The conversion of all-atom structures to CG representations was done using the original MARTINI mapping.

### The Fitness Function for Genetic Algorithm

The fitness function for the development of solvation parameters using Genetic Algorithm was constructed on the basis of changes in geometric parameters of proteins, including the overall change (root-mean-square deviation, RMSD) and changes in the secondary structure elements during MD simulations. Such secondary structure parameters were calculated based on centers of main-chain beads. The fitness function included the following parameters:

- RMSD (root-mean-square deviation)
- Radius of gyration
- A distance between residues *n* and *n*+4 in the α-helix (one helix turn)
- A distance between residues n and *n*+4 in the β-sheet
- A dihedral angle between four consecutive beads of α-helix
- A dihedral angle between four consecutive beads of β-thread

The formula for calculating the fitness function for one protein in shown in eq 7.
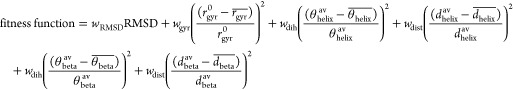
7

The RMSD formula (eq 8) involves a deviation *d*_*i*_ between the current and the initial position of the protein beads, during whole MD simulation, after alignment of protein structures.
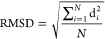
8

The parameter descriptions and values in eq 7 are as follows

- *w*_RMSD_ – weight for RMSD = 1.0 Å^–1^
- *w*_gyr_ – weight for radius of gyration = 3.0
- *w*_dih_ – weight for 1–4 dihedral angles in α-helices or β-sheets = 2.0
- *w*_dist_ – weight for distances between residues *n* and *n*+4 in α-helices or β-sheets = 2.0
- *r*_gyr_^0^ – initial radius of gyration in the crystal structure
- θ_helix_^av^ – all-atom average 1–4 dihedral angle for α-helices = 58.8°
- θ_beta_^av^ – all-atom average 1–4 dihedral angle in β-sheets = 172°
- *d*_helix_^av^ – all-atom average 1–4 distance in α-helices = 5.8 Å
- *d*_beta_^av^ – all-atom average 1–4 distance in β-sheets = 10.3 Å

The fitness function for the whole set of proteins used to develop the solvation parameters was a geometric mean of fitness functions of individual proteins, to not overcome smaller values from some proteins.

### The Genetic Algorithm Procedure

In order to facilitate the parametrization process, we used Genetic Algorithms methodology, a widely recognized class of optimization algorithms inspired by natural selection. The genome of each individual was represented as an array of values to be optimized. For the amino acids, we used 40 coarse-grained bead types for two environments, water and membrane, for solvation parameters: *V*_*i*_, λ_*i*_, Δ*G*_*i*_^ref^, and Δ*G*_*i*_^free^. In total 40 × 2 × 4 = 320 values were to be assessed (Table S1). By grouping similar parameters for similar beads, we obtained 140 parameter values to be optimized. The values were initialized using a discrete set of values uniformly distributed within a range predetermined for each parameter value. The same ranges were used during the mutation stage. Evaluation of each individual required running and analyzing a set of 10 ns molecular dynamics simulations for the training set of proteins. The value of the fitness function for the entire training set of proteins was the geometric mean of the fitness values for all proteins.

The population size of 100 individuals was kept constant and the maximal number of generations was 200. The procedure might have worked either by a predetermined number of generations or until the change in the average fitness value was lower than a given threshold. The children competed with parents during selection for the next generation. We also ensured that each individual in the population was unique and we recorded every genome that appeared during Genetic Algorithm optimization. It was done to prevent running expensive calculations in case any previously eliminated individual reappeared in a later generation as a result of crossover and/or mutation. Probability of mutation was set low at 5%. The parents were selected for mating in a tournament procedure. In brief, a small number of individuals is picked from population and the one with the best fitness function is selected for mating.

The parametrization procedure was implemented in Python 2.7, and we used Distributed Evolutionary Algorithms in Python (DEAP) framework version 1.2.2.^65^ The Scalable COncurrent Operations in Python (SCOOP) module was used for parallelization (repository fork maintained by Institute for Theoretical Computer Science, TU Graz, available at https://github.com/IGITUGraz/scoop). Molecular dynamics simulations using COGRIMEN were run with the modified version of the NAMD program.

### Molecular Dynamics Simulations

MD simulations were performed in NAMD 2.14^66^ with modifications allowing for the introduction of EEF1 and IMM1 as optional methods. With the modified source code, one can simulate system with these methods using parallel computing and GPU, which was not the case in the CHARMM program, where these methods were originally applied. The most intensive parts of the method, calculations of nonbonded interactions consuming a vast majority of processor time, were implemented on GPU to run on CPU/GPU workstations.

The implicit membrane methods, because of their simplicity, could facilitate simulating of large systems in long time scales. Furthermore, a lack of explicit solvent makes it possible to remove a finite periodic simulation box so the computationally intensive calculations of periodic electrostatic (e.g., Ewald summation)^67^ are not necessary. Recent improvements, like addition of membrane dipole potential, make the implicit solvent methods more detailed and reliable.^68^ Usually, these models do not include friction terms, however this problem may be overcome by solving the Langevin equation of motion (eq 9).^69^
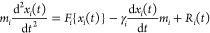
9

In the above formula *F*_*i*_ is a force acting on atom *i*, γ_*i*_ is a friction coefficient, and *R*_*i*_ describes a stochastic motion. We applied Langevin dynamics in 298 K with a damping coefficient of 50 ps^–1^ and a time step of 20 fs. The cutoff with switching for nonbonded interactions (van der Waals and electrostatic) was set at distance 12–14 Å while the cutoff for solvation calculations at 16 Å. For all membrane proteins, we employed thickness of membrane from Orientations of Proteins in Membranes (OPM) database.^60^ In the OPM database each protein is positioned in a lipid bilayer of adjustable thickness by minimizing its transfer energy from water to the membrane, so the membrane thickness is not related to particular lipids. In the COGRIMEN method the membrane thickness is treated as a parameter and can be adjusted to needs, e.g., to simulate the behavior of proteins in the membrane rafts.

### Implementation and Parallelization

The COGRIMEN modifications to the NAMD code are divided into two main parts to avoid overflow of computing and to obtain correct sums/reductions across NAMD spatial decomposition elements called patches. One part is responsible for computing atom by atom, and another for computing pair of atoms by pair of atoms. The first one is written completely separately as additional computing job in each step of simulation. The second one is included in computing of nonbonded interactions using of the existing pair-lists mechanism. The communication is applied by message passing. The second part is definitely the most consuming processing time part and it was the reason for porting it to GPU. Two versions of code are included for running in CPU-only mode and in GPU mode.

Parallelism is done by the Charmm++ programming language/interface, so one can utilize any supported underlying communication mechanism, for example: multicore, Message Passing Interface (MPI), Transmission Control Protocol (TCP), or Infiniband. The GPU version is recommended for longer simulations and/or larger systems. This version is optimized to limit usage of GPU registers, which allow avoidance of overuse of GPU slow local memory. To achieve this goal, IMM1/EEF1 equations are simplified and derived values along with temporary variables are avoided. We also use GPU constant memory and texture fetcher units to utilize GPU cache mechanism.

## Application

### Simulations of Training Set of Proteins

For development of the force field parameters for COGRIMEN, we selected a set of nine proteins (Figure 3). Since the method was developed to study the membrane systems, most of proteins from the training set were transmembrane proteins; however, two of them are cytoplasmic proteins to contribute to development of parameters for the aqueous part of the environment. The proteins contain α-helices, β-sheets, and coil regions, so the parameters were tested in all major types of the secondary structure. For the membrane proteins the helical bundle and the β-barrel proteins are involved, so we do not confine to all-helical membrane proteins, which are the most typical human membrane proteins. All selected proteins are compact, without long and flexible loops, to not artificially increase the RMSD, which is a part of the fitness function. Protein oligomers (trimers) were also included in the training set of proteins to account for protein–protein interactions in parameter values. The transmembrane proteins contain the extramembrane parts, especially for maltoporin (PDB id: 1AF6)^58^ and the membrane anchor domain of the trimeric autotransporter YadA (PDB id: 2LME),^59^ so all selected proteins contribute to development of the solvation parameters both for aqueous and lipid environments.

In Figures S1–S6, we show structures and statistics from 100 ns MD CG simulation using COGRIMEN for training set of proteins: from barstar (PDB id: 1BTA)^51^ to β-barrel platform protein (PDB id: 2JMM).^56^ For all those proteins, we have obtained stable structures, which is confirmed by stable RMSD and radius of gyration (*R*_gyr_) plots. Barstar was the most stable protein with a RMSD below 3 Å (Figure S1). Another cytoplasmic protein, the chemotaxis CheY protein, has a RMSD of about 4.5 Å (Figure S2). The all-helical protein, the isolated voltage-sensing domain containing four transmembrane helices, was very stable with a RMSD of about 3 Å, and the *R*_gyr_ did not change (Figure S3). However, for the CB1 cannabinoid receptor, containing seven transmembrane helices, the RMSD was higher, about 6 Å, and the *R*_gyr_ dropped from 21 to 20 Å, indicating that the resulting structure was slightly more compact (Figure S4). For the next two proteins, being the transmembrane β-barrels, the *R*_gyr_ did not change for both, but the RMSD was 6 Å for the outer membrane protein OmpX (Figure S5) and 11 Å for OmpA (Figure S6). Such a large value of RMSD is a result of thermal movements of a long, flexible extramembrane loops. For all proteins, the histograms showing 1–4 distances and 1–4 dihedral angles of α-helical and β-sheet parts of protein during entire simulation indicate that the secondary elements are stable, and the maximal percentage values correspond to the reference values of these geometric parameters.

The structures and statistics for trimeric proteins are shown in Figures S7–S9. For the bacteriorhodopsin trimer the RMSD is about 8 Å and the *R*_gyr_ did not change and is about 25.4 Å for the trimer. The RMSD for the maltoporin trimer is about 7 Å, and the *R*_gyr_ diminished from 33 to 31 Å, indicating slight compacting of the trimeric structure. For the membrane anchor domain of the trimeric autotransporter YadA the RMSD was 6 Å, and *R*_gyr_ increased from 19.5 to 20.5 Å. The obtained results indicate that the trimers are stable in the long time scales. For all trimers, as it was for the monomeric proteins, the histograms showing 1–4 distances and 1–4 dihedral angles of α-helical and β-sheet parts of protein, indicate that the secondary elements are stable and the maximal percentage values correspond to optimal values of these geometric parameters.

### Simulations of GPCRs with Their Effector Proteins

**Figure 4:**
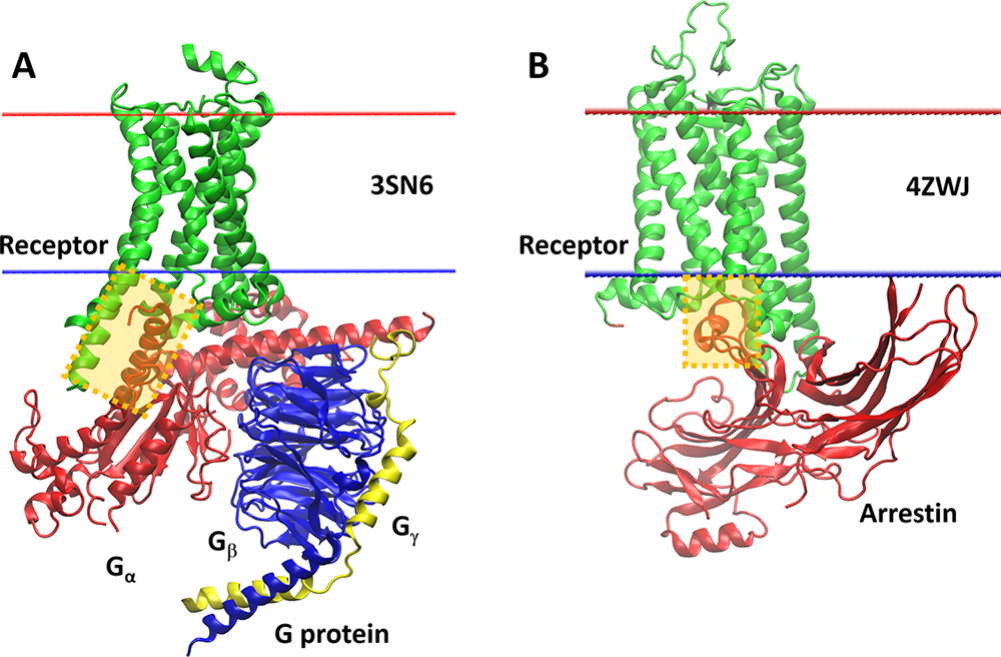
Crystal structures of GPCR complexes with effector proteins. (A) A complex of β2-adrenergic receptor with Gs trimer (G_αβγ_) (PDB id: 3SN6). (B) A complex of rhodopsin with arrestin (PDB id: 4ZWJ). In both panels the receptor is colored in green while the contact between receptor and the effector protein is marked by a semitransparent orange rectangle.

To check whether COGRIMEN could be useful and reliable for investigations of membrane systems different from proteins in the training set, we selected two complexes of GPCRs: a complex of β2-adrenergic receptor with Gs trimer (PDB id: 3SN6) (Figure 4A),^61^ and a complex of rhodopsin with arrestin (PDB id: 4ZWJ) (Figure 4B).^62^ Both complexes are characterized by a small contact area of receptor with the effector protein, so they are vulnerable to structural changes and therefore well suited for verification of the methodology used.

**Figure 5:**
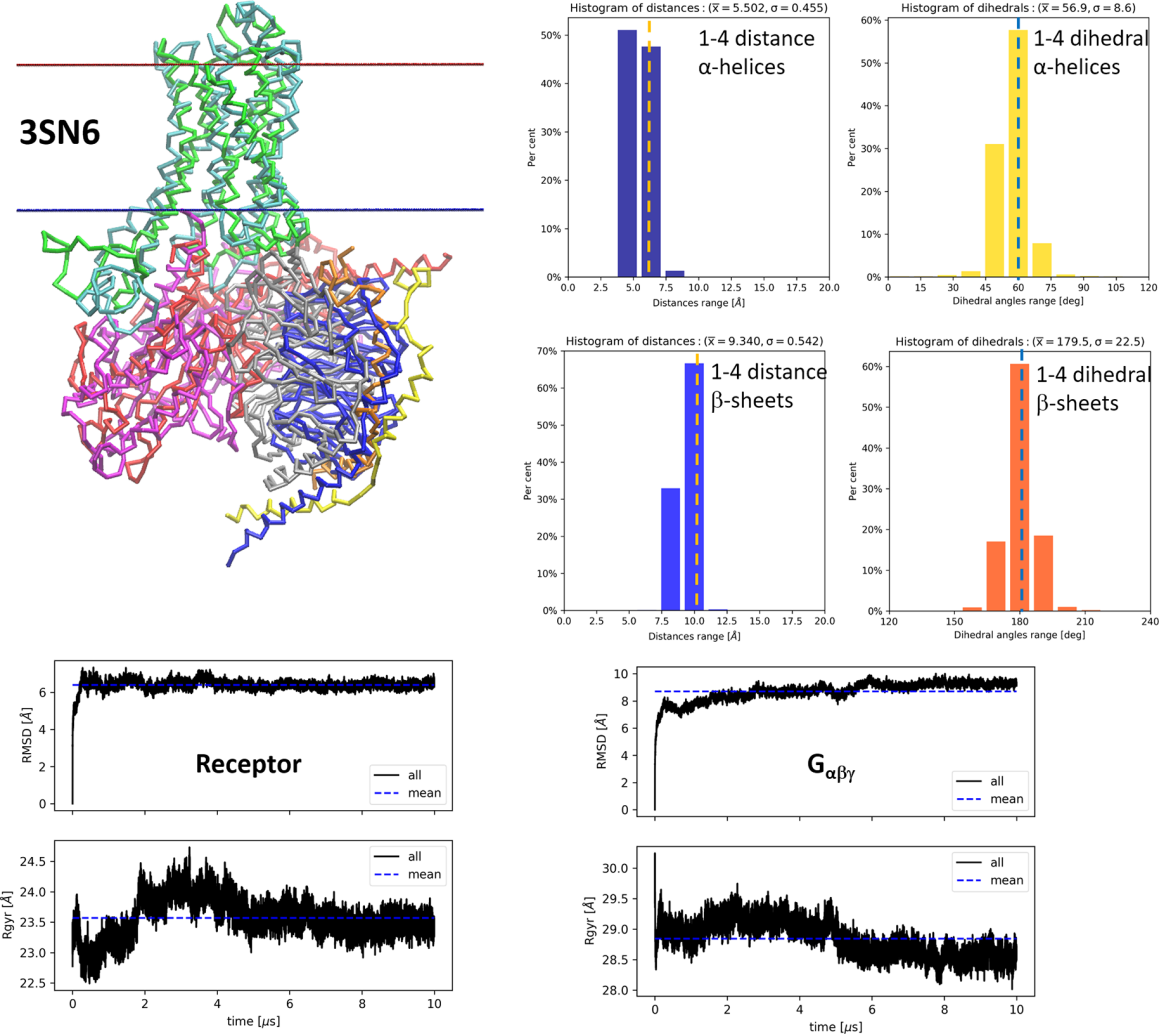
Structures and statistics from 10 μs MD CG simulation of complex of β2-adrenergic receptor with Gs trimer (G_αβγ_) (PDB id: 3SN6). Top-left: superimposition of CG structures, initial (green for receptor, red for G_α_, blue for G_β_, and yellow for G_γ_) and final (cyan for receptor, purple for G_α_, gray for G_β_, and orange for G_γ_). Top-right: histograms of 1–4 distance and 1–4 dihedral angle of α-helical and β-sheet parts of the complex during entire simulation. Bottom: RMSD and radius of gyration plots for receptor, and for trimeric G protein. Dashed vertical lines in histogram plots indicate the reference values for distances and dihedral angles.

For the first complex (PDB id: 3SN6) the superimposition of CG structures indicates that the initial and final structures are similar (Figure 5). In the histograms, showing 1–4 distances and 1–4 dihedral angles of α-helical and β-sheet parts of the whole complex during the entire simulation, the maximal percentage values correspond to optimal values of these geometric parameters, indicating that the secondary elements are stable, as it is for training set of proteins. The RMSD plot for the receptor stabilized at about 6 Å while for trimeric G protein the plot stabilized at a larger value of about 9 Å. This could be a result of movement of the G_α_ subunit, which is composed of two domains, and one of them, not bound directly to the receptor, is highly movable. The radius of gyration (*R*_gyr_) of the receptor changes between 23 and 24 Å and finally stabilizes at 23.5 Å. The same is true for the *R*_gyr_ of the G protein, which stabilized at about 28.5 Å. Regardless of these changes, the final and initial values of the *R*_gyr_ are nearly the same, which might indicate that the overall shape is the same and the protein is not collapsing, a danger for implicit solvent force fields.

**Figure 6:**
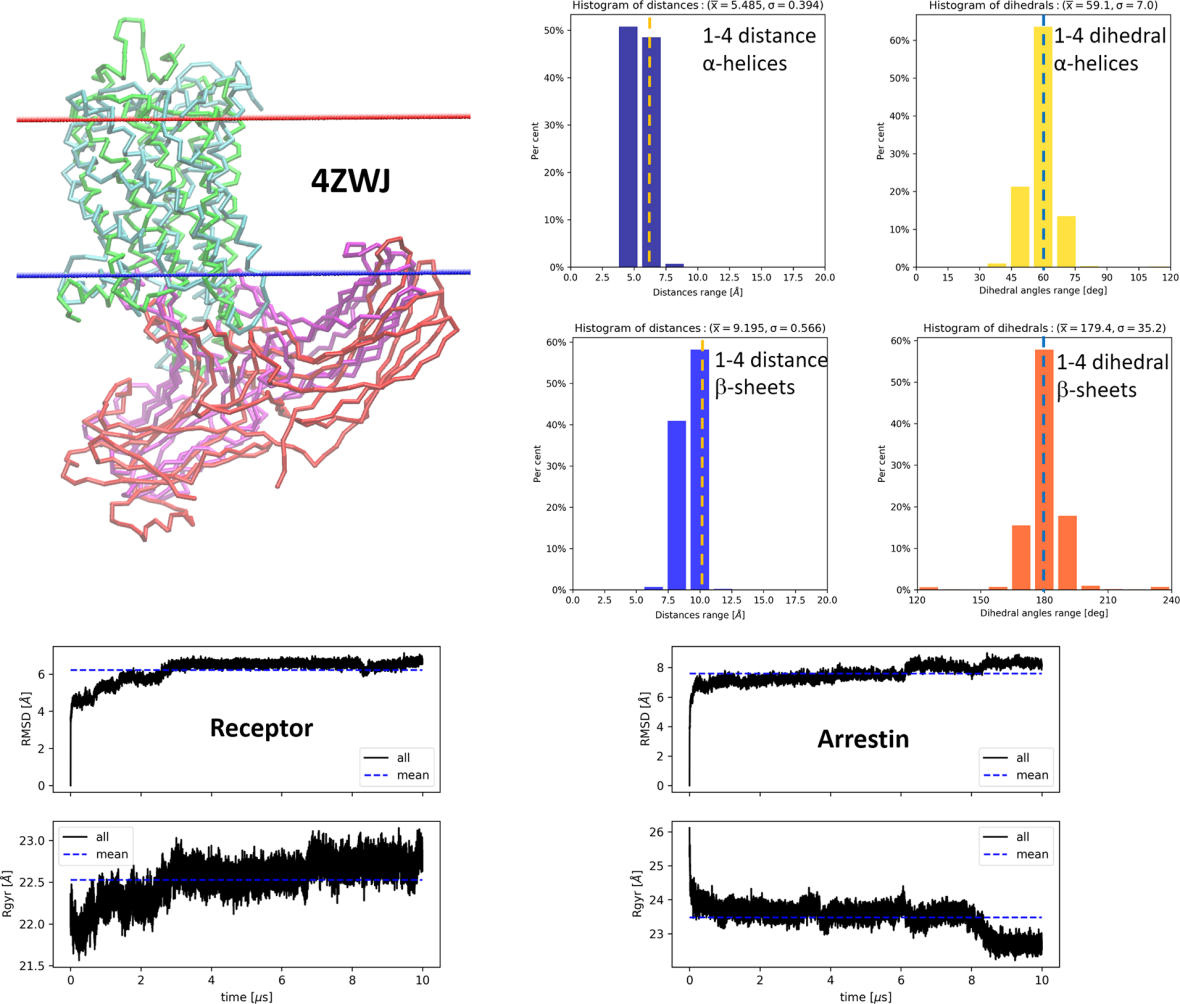
Structures and statistics from 10 μs MD CG simulation of human rhodopsin bound to arrestin (PDB id: 4ZWJ). Top-left: superimposition of CG structures, initial (green for receptor and red for arrestin) and final (cyan for receptor and purple for arrestin). Top-right: histograms of 1–4 distance and 1–4 dihedral angle of α-helical and β-sheet parts of the complex during entire simulation. Bottom: RMSD and radius of gyration plots for receptor and for arrestin. Dashed vertical lines in histogram plots indicate the reference values for distances and dihedral angles.

For the rhodopsin–arrestin complex (PDB id: 4ZWJ) the superimposition of the final and initial CG structures indicates that one part of arrestin prefers to interact with the membrane (Figure 6). Such behavior is implicated by the crystal structure of the complex, which is not symmetrical. The extensive all-atom simulations of this complex also confirmed that this lobe of arrestin is able to bind to the membrane. Such binding is deeper compared to crystal conformation.^70^ The histograms, as for other simulated proteins, indicate that the secondary elements are stable during the entire 10 μs simulation. The RMSD plot for the receptor stabilized at about 6 Å, similarly to the previous complex, while for arrestin it was about 8 Å. The *R*_gyr_ of the receptor increased slightly from 22.0 to 22.7 Å, while for arrestin it diminished from 26 to 23.5 Å at first, and for the last 2 μs to 22.5 Å. Such changes indicate large changes of the shape of arrestin involving independent movements of two lobes of arrestin, which reflect their natural mobility. Arrestin binds tightly to phosphorylated C-termini of GPCRs; however, in the 4ZWJ complex (and also in other complexes with arrestin in PDB) the C-terminus is not visible in the crystal. This could be another source of instability of both arrestin lobes.

## Conclusions

The conducted simulations of training set of proteins, as well as of GPCR complexes with effector proteins, indicate that the proposed methodology is able to reproduce the conformations from crystal structures and is reliable in determining the mobility of loosely coupled parts of the structure: extramembrane loops in the case of training set proteins, and the whole domains in the case of GPCR complexes. COGRIMEN can be useful for fast and preliminary studies of large membrane protein complexes since it represents a simple but reliable methodology that can be used even by nonspecialists. Since MD simulations are becoming increasingly popular to study larger and larger complexes to reveal their dynamic behavior and crowding phenomena, the usage of COGRIMEN could be a valuable option.

## Supporting Information Available

(Figures S1–S6) Structures and statistics of 100 ns MD CG simulation using COGRIMEN for training set proteins–monomers; (Figures S7–S9) Structures and statistics of 10 μs MD CG simulation using COGRIMEN for training set proteins–trimers; (Table S1) Values of solvation parameters developed for MARTINI bead types in the COGRIMEN method (PDF)

## Accession Codes

The code of COGRIMEN 1.0 patch together with build notes for NAMD 2.14, the parameter files, and the example input files are deposited on the Web site https://cogrimen.biomodellab.eu/
