# Supplementary material for: COGRIMEN: Coarse-Grained Method for Modeling of Membrane Proteins in Implicit Environments
Source: J Chem Theory Comput. 2022 Aug 23;18(9):5145–56. doi: 10.1021/acs.jctc.2c00140 (PMC9476660; doi:10.1021/acs.jctc.2c00140)
Supplement: Supplementary file 1 — ct2c00140_si_001.pdf [file ct2c00140_si_001.pdf]

Supporting Information

COGRIMEN – Coarse-Grained Method for Modeling of Membrane Proteins in Implicit Environments

Przemysław Miszta[1#], Paweł Pasznik[1#], Szymon Niewieczerzał[1#], Krzysztof Młynarczyk[1], Sławomir Filipek[1*]

[1] Faculty of Chemistry, Biological and Chemical Research Centre, University of Warsaw, Warsaw, Poland.

[#] These authors contributed equally to the work

[*] corresponding author, e-mail: sh.filipek@uw.edu.pl

Supporting figures

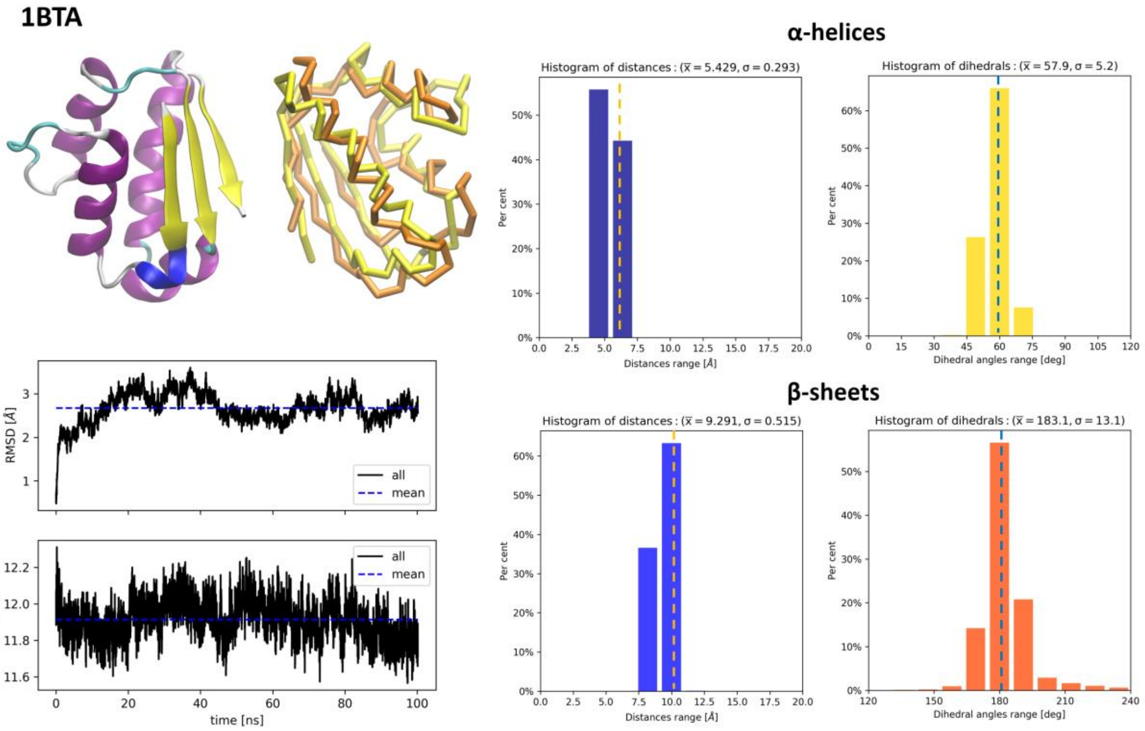

Figure S1. Structures and statistics of 100 ns MD CG simulation using COGRIMEN for training set protein – barstar (PDB id:1BTA). Up-left: all-atom structure and superimposition of CG structures initial (yellow) and final (orange); down-left: RMSD and radius of gyration plots; up-right: histograms of 1–4 distance and 1–4 dihedral angle of α-helical part of protein; down-right: histograms of 1–4 distance and 1–4 dihedral angle of β-sheet part of protein. Dashed vertical lines in histogram plots indicate the reference values for distances and dihedral angles.

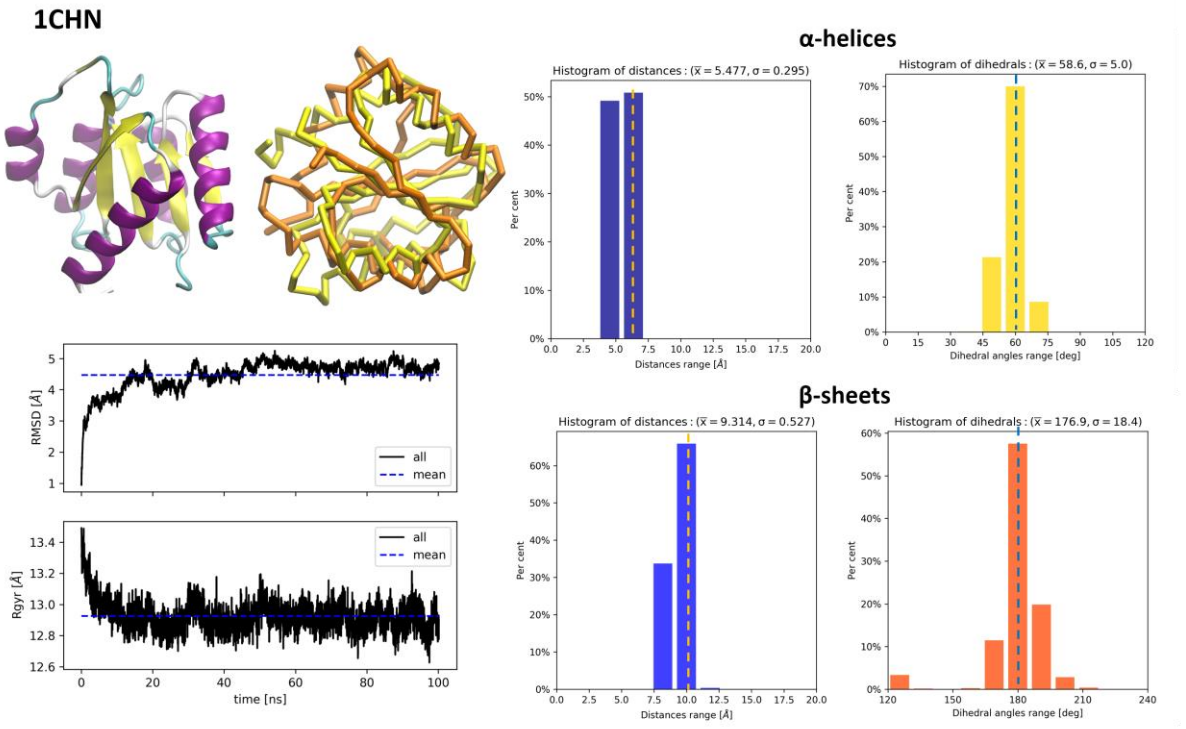

Figure S2. Structures and statistics of 100 ns MD CG simulation using COGRIMEN for training set protein –bacterial chemotaxis protein CheY (PDB id:1CHN). Up-left: all-atom structure and superimposition of CG structures initial (yellow) and final (orange); down-left: RMSD and radius of gyration plots; up-right: histograms of 1–4 distance and 1–4 dihedral angle of α-helical part of protein; down-right: histograms of 1–4 distance and 1–4 dihedral angle of β-sheet part of protein. Dashed vertical lines in histogram plots indicate the reference values for distances and dihedral angles.

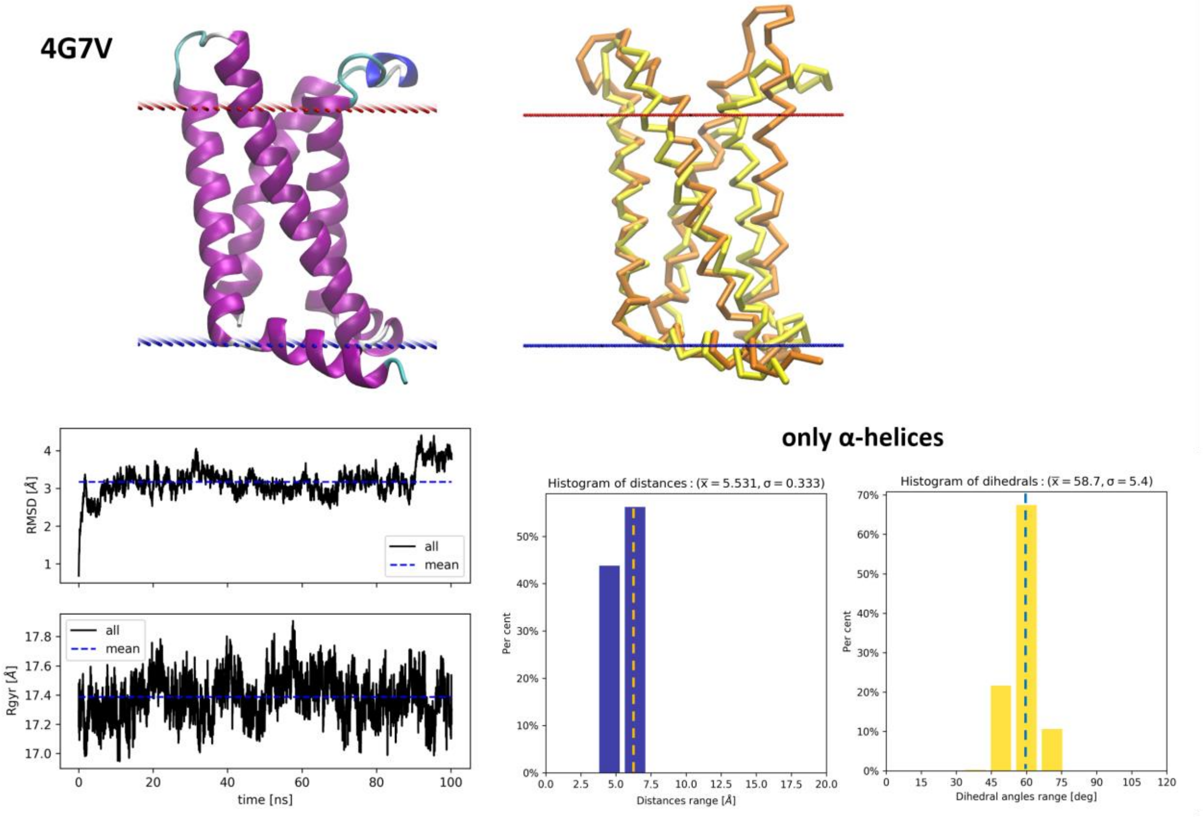

Figure S3. Structures and statistics of 100 ns MD CG simulation using COGRIMEN for training set protein – 4TM isolated voltage-sensing domain (PDB id:4G7V). Up: all-atom structure and superimposition of CG structures initial (yellow) and final (orange); down-left: RMSD and radius of gyration plots; down-right: histograms of 1–4 distance and 1–4 dihedral angle of α-helical part of protein. Dashed vertical lines in histogram plots indicate the reference values for distances and dihedral angles.

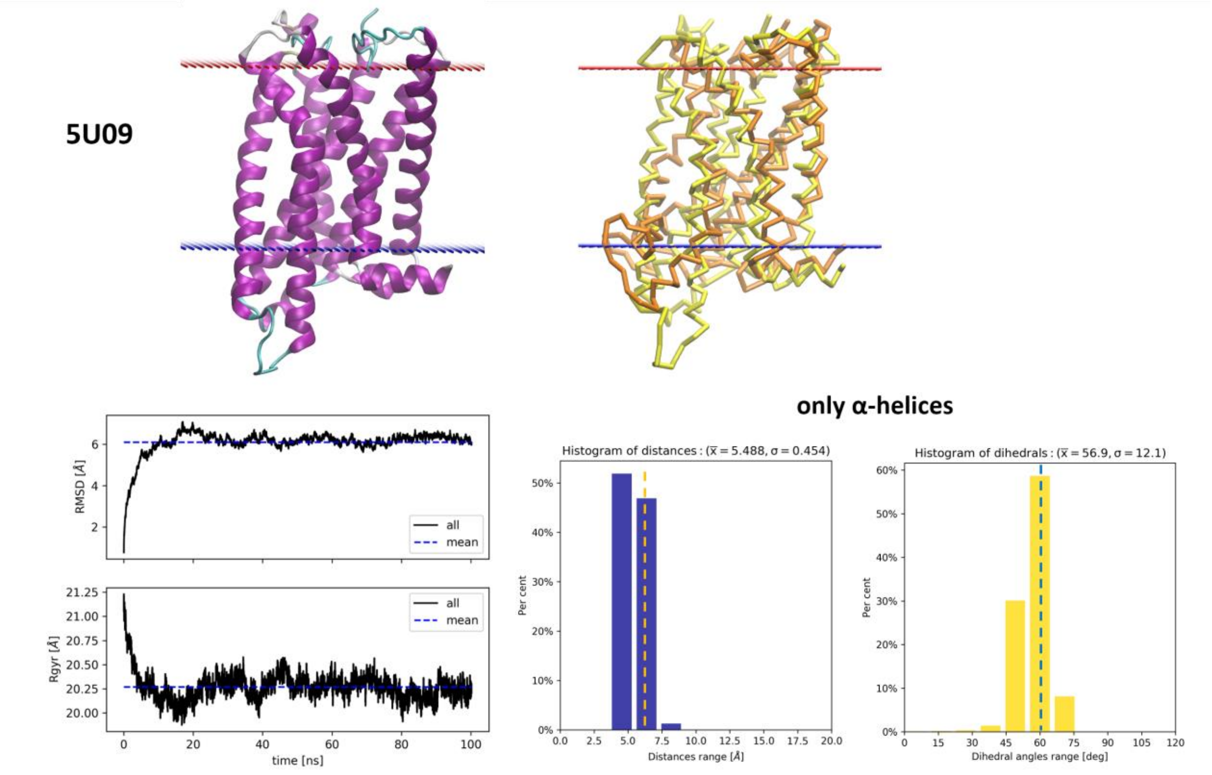

Figure S4. Structures and statistics of 100 ns MD CG simulation using COGRIMEN for training set protein – 7TM CB1 cannabinoid receptor (PDB id:5U09). Up: all-atom structure and superimposition of CG structures initial (yellow) and final (orange); down-left: RMSD and radius of gyration plots; down-right: histograms of 1–4 distance and 1–4 dihedral angle of α-helical part of protein. Dashed vertical lines in histogram plots indicate the reference values for distances and dihedral angles.

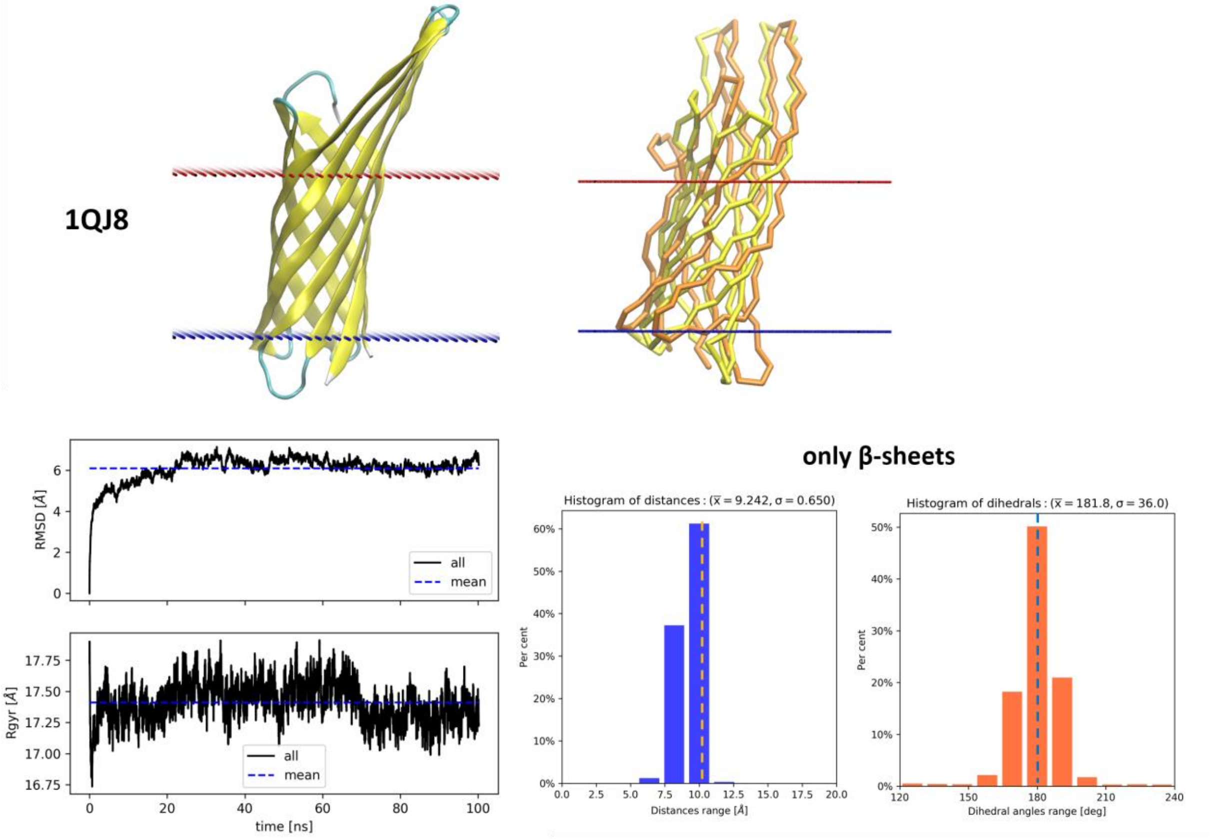

Figure S5. Structures and statistics of 100 ns MD CG simulation using COGRIMEN for training set protein – the outer membrane protein OmpX (PDB id:1QJ8). Up: all-atom structure and superimposition of CG structures initial (yellow) and final (orange); down-left: RMSD and radius of gyration plots; down-right: histograms of 1–4 distance and 1–4 dihedral angle of β-sheet part of protein. Dashed vertical lines in histogram plots indicate the reference values for distances and dihedral angles.

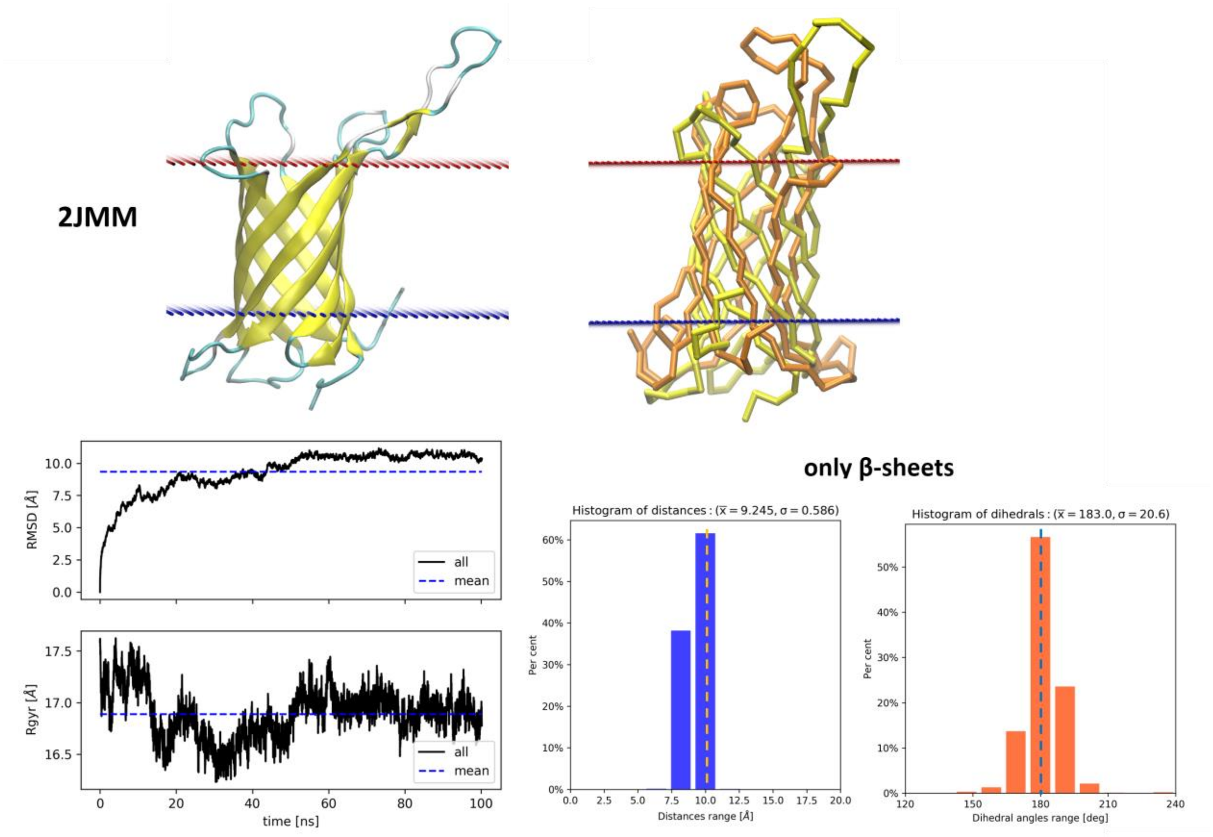

Figure S6. Structures and statistics of 100 ns MD CG simulation using COGRIMEN for training set protein – the outer membrane protein OmpA (PDB id:2JMM). Up: all-atom structure and superimposition of CG structures initial (yellow) and final (orange); down-left: RMSD and radius of gyration plots; down-right: histograms of 1–4 distance and 1–4 dihedral angle of β-sheet part of protein. Dashed vertical lines in histogram plots indicate the reference values for distances and dihedral angles.

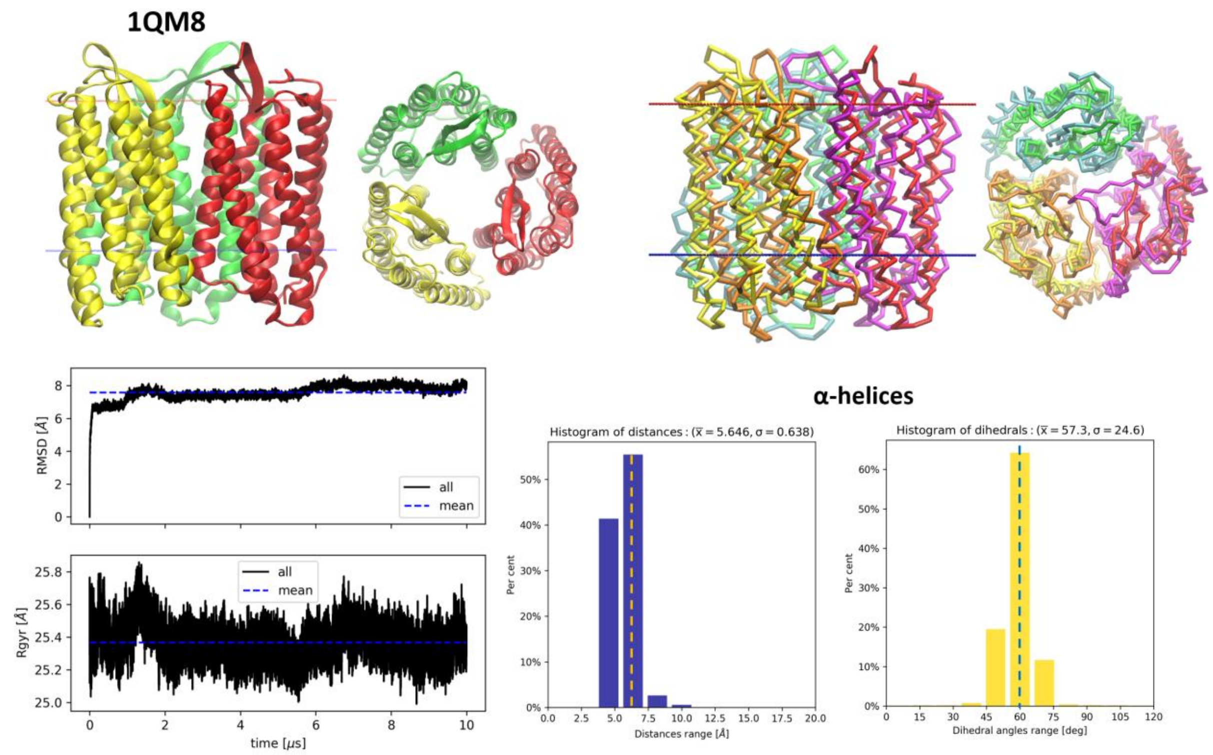

Figure S7. Structures and statistics of 10 μs MD CG simulation using COGRIMEN for training set protein – trimer of bacteriorhodopsin from *Halobacterium salinarum* (PDB id:1QM8). Up-left: all-atom structure, side and top views; up-right: superimposition of CG structures initial (yellow, red and green) and final (orange, purple and cyan), side and top views; down-left: RMSD and radius of gyration plots; down-right: histograms of 1–4 distance and 1–4 dihedral angle of α-helical part of protein. Dashed vertical lines in histogram plots indicate the reference values for distances and dihedral angles.

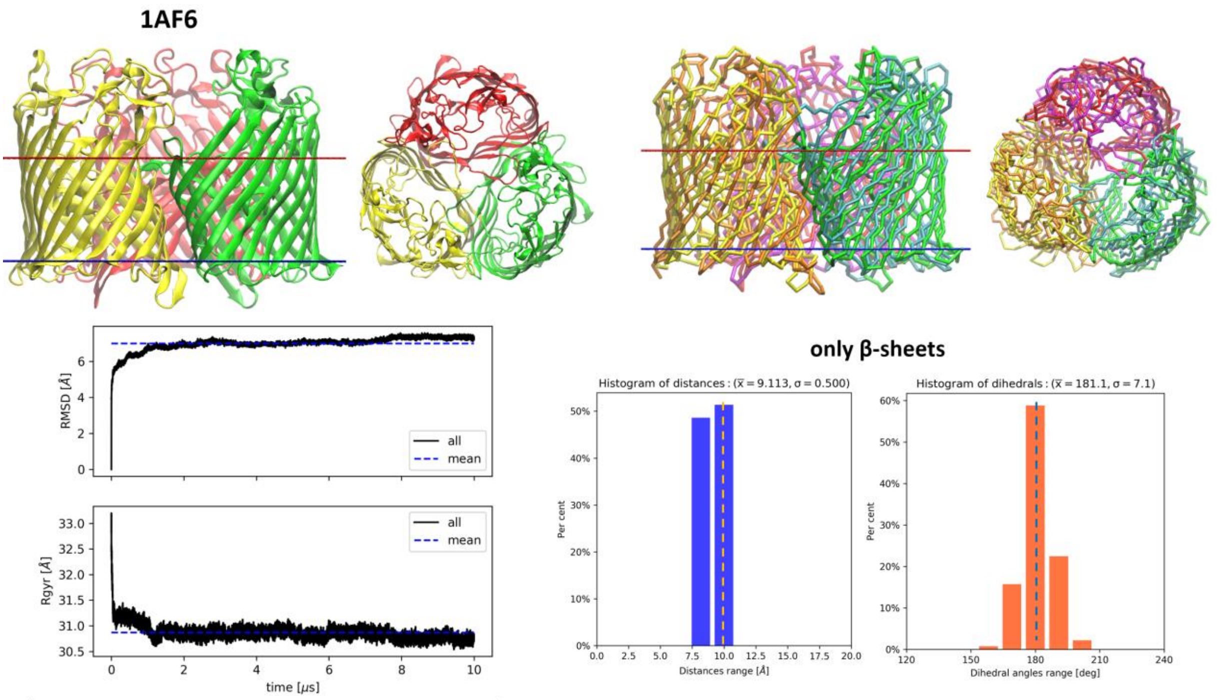

Figure S8. Structures and statistics of 10 μs MD CG simulation using COGRIMEN for training set protein – trimer of maltoporin from *Escherichia coli* (PDB id:1AF6). Up-left: all-atom structure, side and top views; up-right: superimposition of CG structures initial (yellow, red and green) and final (orange, purple and cyan), side and top views; down-left: RMSD and radius of gyration plots; down-right: histograms of 1–4 distance and 1–4 dihedral angle of β-sheet part of protein. Dashed vertical lines in histogram plots indicate the reference values for distances and dihedral angles.

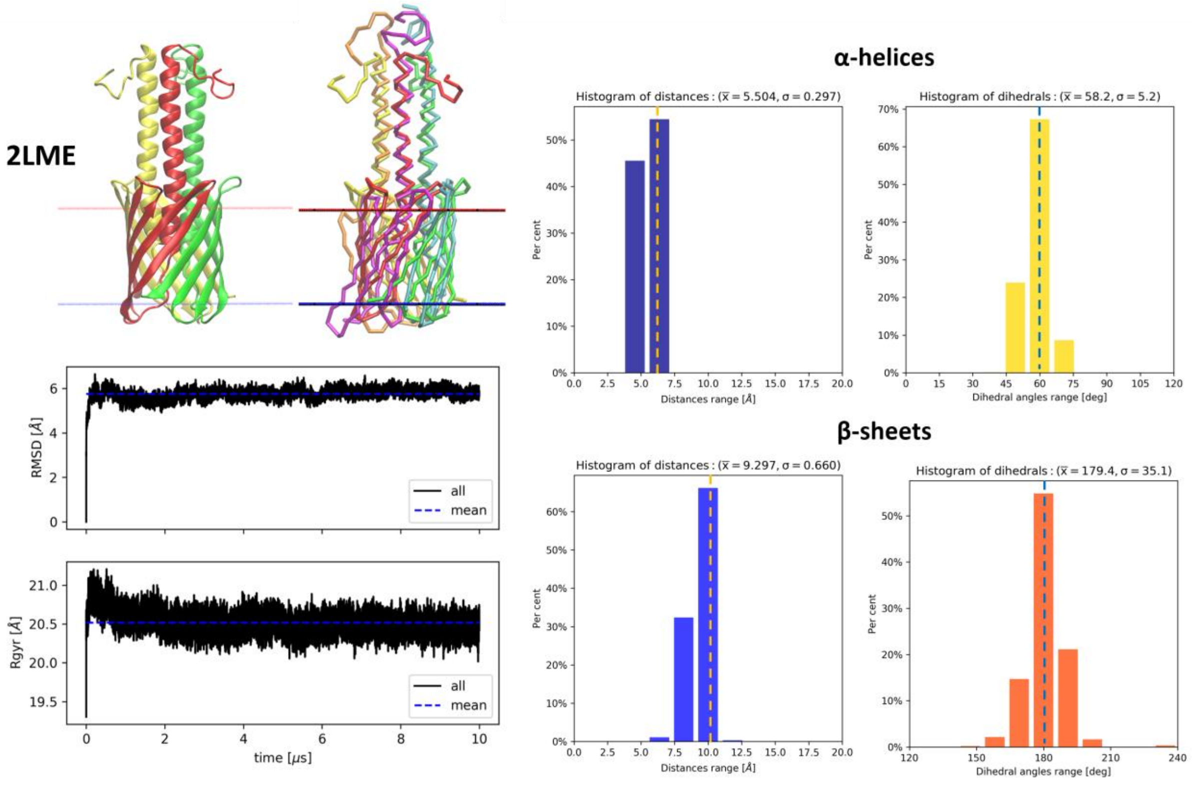

Figure S9. Structures and statistics of 10 μs MD CG simulation using COGRIMEN for training set protein – the membrane anchor domain of the trimeric autotransporter YadA from *Yersinia enterocolitica* (PDB id:2LME). Up-left: all-atom structure and superimposition of CG structures initial (yellow, red and green) and final (orange, purple and cyan), side views; down-left: RMSD and radius of gyration plots; up-right: histograms of 1–4 distance and 1–4 dihedral angle of α-helical part of protein; down-right: histograms of 1–4 distance and 1–4 dihedral angle of β-sheet part of protein. Dashed vertical lines in histogram plots indicate the reference values for distances and dihedral angles.

Supporting tables

Table S1. Values of solvation parameters developed for MARTINI bead types in COGRIMEN method.

Bead type	V_i	λ_i	ΔG_i^{ref}	ΔG_i^{free}
**In membrane**				
NAB	37.66	4.4	-9.405	-5.768
NAC	37.66	2	-9.405	-5.768
AC2P	70.56	12	-4.471	-4.666
SP1Y	28.69	12	-5.984	-7.921
AC2V	58.59	12	-3.357	-2.916
N0HP	37.66	14.4	-9.405	-5.768
NAH	37.66	14.4	-9.405	-5.768
P4C	37.66	1.8	-9.405	-5.768
P4B	37.66	4.4	-9.405	-5.768
QAE	51.09	12	-8.456	-5.730
QAD	49.89	12	-3.881	-6.744
P4Q	134.47	12	-7.710	-7.777
C5HP	37.66	17.6	-9.405	-5.768
SQDH	15.05	12	-5.213	-3.656
QDR	40.06	12	-1.925	-6.376
SC4H	31.77	12	-5.920	-7.065
NDH	37.66	14.4	-9.405	-5.768
P5N	82.74	12	-6.772	-7.272
N0H	37.66	17.6	-9.405	-5.768
N0E	37.66	1.8	-9.405	-5.768
P3C	61.19	2.2	-6.489	-12.236
SC4F	20.25	12	-3.059	-2.609
P5B	37.66	4.4	-9.405	-5.768
SP1W	33.06	12	-2.524	-4.809
SC4Y	47.25	12	-4.785	-4.799
P1T	26.59	12	-2.122	-3.685
P5C	37.66	2.2	-9.405	-5.768
N0T	37.66	2.2	-9.405	-5.768
AC1L	53.05	12	-6.549	-8.162
SC4W	27.93	12	-6.515	-7.803
AC1I	53.05	12	-6.549	-8.162
N0R	74.11	12	-10.542	-8.550
NDAE	37.66	2.2	-9.405	-5.768
C3K	70.56	12	-4.471	-4.666
QDK	27.22	12	-4.591	-3.456
NDAT	37.66	2.2	-9.405	-5.768
P1S	36.52	12	-2.873	-3.234
C5C	43.36	12	-6.438	-5.589
C5M	132.9	12	-9.440	-6.851

C5H	37.66	16	-9.405	-5.768
**In water**	V_i	λ_i	ΔG_i^{ref}	ΔG_i^{free}
NAB	37.66	4.4	-11.999	-15.653
NAC	37.66	2.2	-11.999	-15.653
AC2P	70.56	12	1.562	4.368
SP1Y	28.69	12	-13.795	-22.456
AC2V	58.59	12	4.181	7.260
N0HP	37.66	14.4	-11.999	-15.653
NAH	37.66	14.4	-11.999	-15.653
P4C	37.66	2.2	-11.999	-15.653
P4B	37.66	4.4	-11.999	-15.653
QAE	51.09	12	-55.073	-33.370
QAD	49.89	12	-23.75	-51.622
P4Q	134.47	12	-9.936	-35.211
C5HP	37.66	17.6	-11.999	-15.653
SQDH	15.05	12	-8.256	-19.075
QDR	40.06	12	-30.082	-46.774
SC4H	31.77	12	-1.367	-2.278
NDH	37.66	14.4	-11.999	-15.653
P5N	82.74	12	-18.734	-43.329
N0H	37.66	17.6	-11.999	-15.653
N0E	37.66	2.2	-11.999	-15.653
P3C	61.19	2.2	-11.999	-42.321
SC4F	20.25	12	-0.203	-0.313
P5B	37.66	4.4	-11.999	-15.653
SP1W	33.06	12	-5.304	-27.93
SC4Y	47.25	12	-0.318	-0.425
P1T	26.59	12	0.902	2.200
P5C	37.66	2.2	-11.999	-15.653
N0T	37.66	2.2	-11.999	-15.653
AC1L	53.05	12	6.639	7.063
SC4W	27.93	12	-1.724	-2.929
AC1I	53.05	12	6.639	7.063
N0R	74.11	12	-5.823	-8.287
NDAE	37.66	2.2	-11.999	-15.653
C3K	70.56	12	1.562	4.368
QDK	27.22	12	-23.554	-42.856
NDAT	37.66	2.2	-11.999	-15.653
P1S	36.52	12	-7.324	-18.603
C5C	43.36	12	-5.168	-4.528
C5M	132.9	12	-1.407	-1.901
C5H	37.66	16	-11.999	-15.653

Units: V_i [Å], λ_i [Å], ΔG_i^{ref} [kcal/mol], ΔG_i^{free} [kcal/mol].
